# Genome Sequence of a Recombinant SARS-CoV-2 Lineage XAM (BA.1.1/BA.2.9) Strain from a Clinical Sample in Santo Domingo, Dominican Republic

**DOI:** 10.1128/mra.01113-22

**Published:** 2022-11-30

**Authors:** Robert Paulino-Ramírez, Kien Pham, Mallery I. Breban, Armando Peguero, Maridania Jabier, Nelissa Sánchez, Iscania Eustate, Ingrid Ruiz, Nathan D. Grubaugh, Anne M. Hahn

**Affiliations:** a Instituto de Medicina Tropical y Salud Global, Universidad Iberoamericana, UNIBE Research Hub, Santo Domingo, Dominican Republic; b Department of Epidemiology of Microbial Diseases, Yale School of Public Health, New Haven, Connecticut, USA; c Servicio Nacional de Salud, Ministry of Health, Santo Domingo, Dominican Republic; Queens College CUNY

## Abstract

Here, we report a recombinant severe acute respiratory syndrome coronavirus 2 (SARS-CoV-2) lineage XAM (Omicron BA.1.1/BA.2.9) strain that was collected in Santo Domingo, Dominican Republic. This demonstrates how SARS-CoV-2 variants can vary greatly between regions and thus underlines the great importance of regional genomic surveillance efforts.

## ANNOUNCEMENT

The betacoronavirus (*Coronaviridae*) severe acute respiratory syndrome coronavirus 2 (SARS-CoV-2), the causative agent of coronavirus disease 2019 (COVID-19), remains a major public health threat despite the availability of vaccines and therapeutics. Since late 2020, a continuing surge of SARS-CoV-2 variants has been observed globally. The currently dominant variant, Omicron, was first identified in November 2021 (Pango lineage B.1.1.529/BA.1) and has since continued to diversify in various sublineages (1, 2). The generation of this and other variants of concern likely involves mechanisms including accelerated evolution in chronically infected hosts and recombination during coinfections with different variants (3–5).

During high transmission of cocirculating SARS-CoV-2 variants, coinfections might lead to genomic recombination, which plays an important evolutionary role in advantageous gene transfer, phenotypic modifications, and anthroponotic-zoonotic adaptations (6). Within Omicron, several recombinant events have been recognized. For example, XE (BA.1/BA.2) reached an ~10% increase in transmissibility, compared to BA.2 subvariants (7, 8). In October 2022, XBB, a BA.2.10 and BA.2.75 recombinant, reached dominance in Singapore (https://cov-spectrum.org/explore/Singapore/AllSamples/Y2022/variants?variantQuery=nextcladePangoLineage%3AXBB*&).

**FIG 1:**
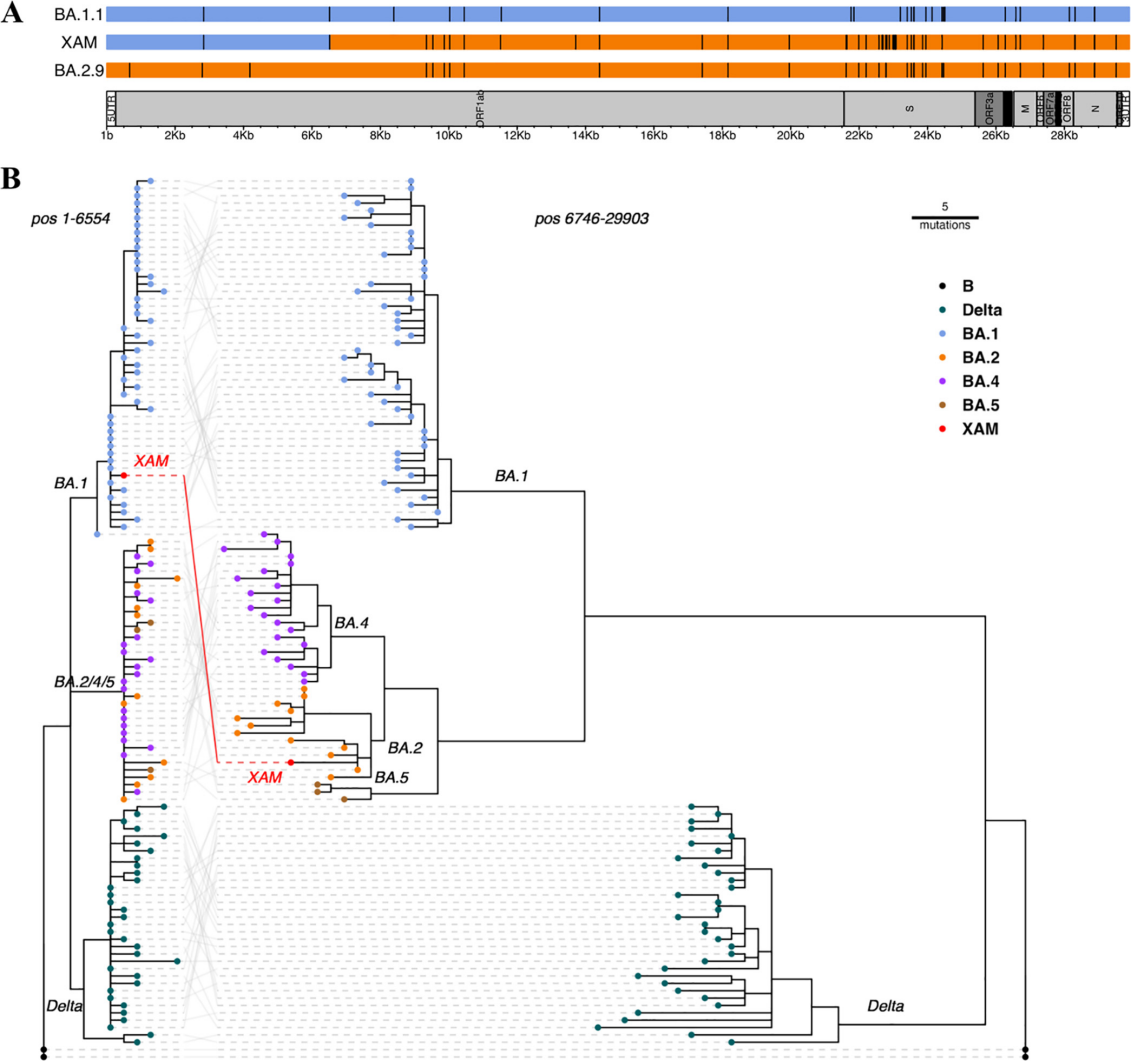
SARS-CoV-2 recombinant XAM strain detected in the Dominican Republic genomic surveillance. (A) Cartoon of the parental lineages BA.1.1 and BA.2.9 in comparison with XAM. (B) Tangle-gram depicting phylogenies of SARS-CoV-2 genomes split at the recombination breakpoint with samples from the Dominican Republic. The phylogenetic tree was built using Nextstrain (17). Samples were randomly selected from all Dominican Republic sequences submitted by the Grubaugh laboratory to subsample a set of 10 genomes for every month since November 2021. The json file generated was visualized with R package ggtree (18). The lengths of the branches represent phylogenetic distances from the reference genome.

In the Dominican Republic, a rapid influx of variants that had been previously reported in countries with cultural and commercial ties was observed after easing of lockdowns and reopening of the tourist industry (9, 10). Here, we report the detection of SARS-CoV-2 lineage XAM, a BA.1.1 and BA.9 recombinant (Fig. 1) that was first identified in March 2022. The sequence was obtained from a nasopharyngeal swab sample taken on 21 June 2022 from a 43-year-old symptomatic resident in the capital city, Santo Domingo. RNA extraction was performed following the protocol provided by the IVD RADI PREP swab and stool DNA/RNA extraction kit (KH Medical Co., Republic of Korea). The sample was confirmed to be SARS-CoV-2 positive (cycle threshold [*C_T_*] value of 26) by real-time quantitative PCR (RT-qPCR) using the virellaSARS-CoV-2 seqc amplification protocol (gerbion GmbH & Co., Germany). Amplicon-based library preparation was performed with the Illumina COVIDseq research use only (RUO) test and ARTIC v.4.1 primers (Integrated DNA Technologies). Sequencing was performed on an Illumina NovaSeq 600 system (2 × 150-bp, paired-end reads), with an average read length of 133 bp. For sequence data analysis, the tools listed in Table 1 were used with default parameters unless otherwise specified. Raw reads were processed with the Illumina bcl2fastq pipeline (v.2.20.0) and BWA-MEM (v.0.7.15) (11) using Wuhan-Hu-1 as the reference genome (GenBank accession MN908947.3). A consensus sequence was built using iVar (v.1.3.1) (12) and SAMtools (v.1.7) (13). Lineage assignment according to the Pangolin nomenclature (10) was conducted with Nextclade (v.2.8.0) (14). This reported sequence has a genome size of 29,740 bp, a total of 2,834,709 raw reads, with a GC content of 39.18%, and a SARS-CoV-2 coverage depth of 14,219.52×.

**TABLE 1:** Software used for sequence analysis

Name	Source	URL	Reference
R	CRAN	https://www.R-project.org	
ggtree	GitHub	https://github.com/YuLab-SMU/ggtree	18
Augur	GitHub	https://github.com/nextstrain/augur	17
SAMtools	GitHub	https://samtools.github.io	13
iVar	GitHub	https://github.com/andersen-lab/ivar	12
BWA-MEM	GitHub	https://github.com/lh3/bwa	11
Pangolin	GitHub	https://github.com/cov-lineages/pangolin	19
bcl2fastq	Illumina	https://support.illumina.com/downloads/bcl2fastq-conversion-software-v2-20.html	
Nextclade	Github	https://github.com/nextstrain/nextclade	14
Nextstrain	Github	https://github.com/nextstrain/nextstrain.org	17
Subsampler	Github	https://github.com/andersonbrito/subsampler	20

This study was approved by the Universidad Iberoamericana (UNIBE) institutional review board (approval number CEI-2020-16), the National Bioethical Committee (CONABIOS) (approval number 020-2021), and the institutional review board of the Yale University Human Research Protection Program (approval number 2000031374).

Especially in island nation settings, the clinical and epidemiological relevance of these new genetic combinations is substantial, since they may provoke the emergence of autochthonous variants (15, 16). With continuing high global transmission rates, inequitable vaccine access and coverage and numerous long-term infections in immunosuppressed hosts present high risks for emerging SARS-CoV-2 variants. Thus, local and regional genomic surveillance should be continued and monitoring networks strengthened.

### Data availability.

The sequence of the SARS-CoV-2 recombinant variant was deposited in the GISAID database with accession number EPI_ISL_1509635 and in GenBank with accession number OP682879. The raw read data were deposited in the NCBI Sequence Read Archive (SRA) with BioProject accession number PRJNA891871 and SRA accession number SRR21966550.
